# Spontaneous recurrent episodes of wrist pain in a 16-year-old girl: a case of complex regional pain syndrome

**DOI:** 10.1097/PR9.0000000000000578

**Published:** 2016-11-30

**Authors:** Janne Gierthmühlen, Ralf Baron, Markus Blankenburg, Boris Zernikow, Christoph Maier

**Affiliations:** a Division of Neurological Pain Research and Therapy, Department of Neurology, Universitätsklinikum Schleswig-Holstein, Kiel, Germany; b Pediatric Neurology, Psychosomatics and Pain Therapy, Center for Child, Youth and Women's Health, Klinikum Stuttgart, Olgahospital/Frauenklinik, Stuttgart, Germany; c Vodafone Foundation Institute for Children's Pain Therapy and Paediatric Palliative Care, Children's and Adolescents' Hospital Datteln, Witten/Herdecke University, Germany; d Department of Pain Management, Berufsgenossenschaftliches Universitätsklinikum Bergmannsheil, GmbH Bochum, Ruhr University Bochum, Bochum, Germany

**Keywords:** CRPS (complex regional pain syndromes), Reflex sympathetic dystrophy, Child, Pain

## Abstract

**Introduction:** Complex regional pain syndromes (CRPS) are disabling pain syndromes that can develop after minor tissue injury or trauma and are characterized by sensory, motor, and autonomic abnormalities distributed in a glove-like or stocking-like manner. Complex regional pain syndrome is well known in adults, but is relatively rare in children. Most of the reported cases of CRPS in children are clinical diagnoses that are not supported by examinations such as three-phase bone scintigraphy. Furthermore, different centres often use different diagnostic criteria for CRPS, which sometimes questions the diagnosis of CRPS.

**Objective/Methods:** Although, recurrences and in particular relapses of CRPS have been observed, a periodically, nearly self-limiting course of disease without any residues in pain-free episodes and without any new obvious injury in CRPS is rather unusual. We present the case of a 16-year-old girl who reported recurrent spontaneous pain which lasted for 2 to 3 weeks and occurred approximately 2 times a year after the patient had experienced a mild trauma 3 years ago.

**Results:** The pain was accompanied by swelling, temperature asymmetry, and decreased range of motion of the right hand without any complains in pain-free episodes. Recurrent symptoms occurred without any obvious trauma. Diagnosis of CRPS was made from clinical findings including quantitative sensory testing, increased periarticular radioisotope uptake in the delayed phase of three-phase bone scintigraphy and examination during anaesthesia. Multimodal inpatient pain treatment resolved her symptoms substantially.

**Conclusion:** Complex regional pain syndrome in children may imitate rheumatologic diseases, and the course is different from adults despite similar clinical findings.

## Abstract 2

CRPS in children may imitate rheumatologic diseases, but should be considered if typical signs are present. Diagnosis can be supported by three-phase bone scintigraphy.

## 1. Introduction

Complex regional pain syndrome (CRPS) is characterized by sensory, motor, and autonomic abnormalities that typically show a generalized distal distribution.^2^ Fractures and sprains are the most common trigger preceding CRPS, whereas a spontaneous onset is rare.^5^ Residues of the disease usually persist for a long time.^4,14^

Diagnosis of CRPS in children may be delayed because of the low incidence and a slightly different clinical presentation compared with adults including a more commonly affected lower limb, a less frequent severe trauma, less pronounced neurological and autonomic signs, and a greater role of psychological and social factors.^11,18^ Most of the reported cases of CRPS in children are clinical diagnoses and not supported by examinations such as three-phase bone scintigraphy (TPBS).^20^

### 1.1. Case

A 16-year-old girl presented with 5 weeks of swelling and spontaneous pain of the right wrist and hand with an intensity of 8 on the numerical rating scale (0 = no pain and 10 = strongest pain imaginable). She also felt the right hand was warmer than the left. Light touch, heat, cold, and every movement or exposure would cause pain. She had not experienced any injury preceding the onset of symptoms. Three years ago, she had fallen on the extended hand. Since then she had been suffering from short episodes of pain lasting 2 to 3 weeks approximately 2 times a year. These episodes presented with spontaneous pain, temperature asymmetries, pain from light touch or cold, and a decreased range of motion, although not as intense in symptoms and long in duration as the current episode. In between the pain episodes, she had not observed any abnormalities and no problems using her right arm for her hobbies (riding, volleyball, dancing, and writing), although she reported that she sometimes had experienced mild pain and swelling after strong exposure. During the former episodes, she had been treated with oral and topical nonsteroidal anti-inflammatory drug as well as physiotherapy and sometimes also received a temporary splint. After this treatment, the symptoms always resolved. Several x-ray examinations and magnetic resonance tomographies of the right hand had been performed during and after the recurrent pain episodes as well as during the current episode had never shown any specific pathology. Former and current routine laboratory tests including erythrocyte sedimentation rate, C-reactive protein, antistreptolysin, rheumatoid factor, and serotest for borreliosis were normal as were median and ulnar nerve neurography.

**Figure 1.**
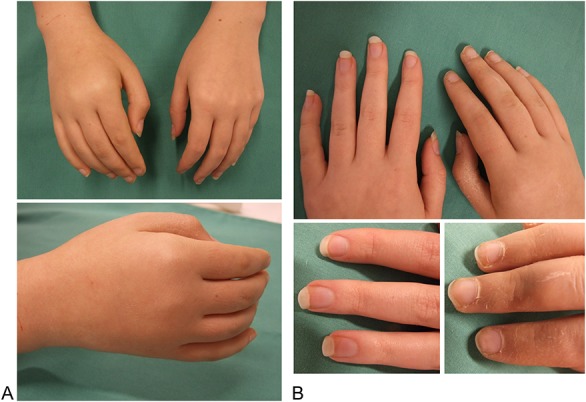
Autonomic clinical findings. (A) Slight swelling in a glove-like manner on the affected right hand. (B) Figure showing trophic disturbances on the affected right extremity with dry skin and a reduced nail growth. Lower figures: left, left healthy extremity; right, affected extremity.

On examination, she presented with a mild swelling, reddish skin color, and dry skin on the right hand. Nail growth differed between right and left hand with the patient reporting that nail growth was slower on the affected right hand (Fig. 1). There were no temperature differences >1°C compared with the left hand.

**Figure 2.**
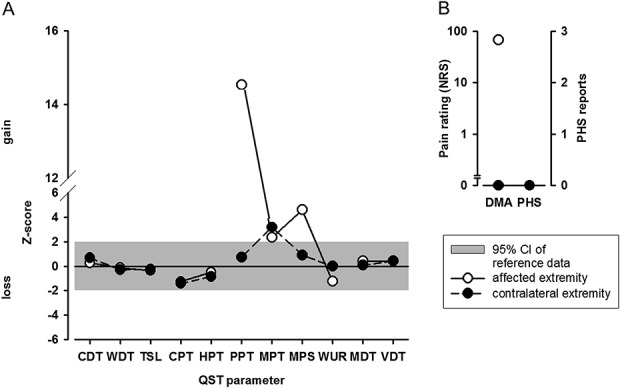
Somatosensory profile of the affected and corresponding contralateral extremity. Figure shows calculated z-values based on a reference database of healthy controls.^15^ Z-values indicate hypofunction or hyperfunction of the subject's sensitivity for each parameter as compared with the mean of age-matched and gender-matched controls. The 95% CI of controls is between −1.96 and +1.96 (light blue area). Z-values above “0” indicate hyperfunction, ie, patients are more sensitive to the tested parameter compared with controls (lower thresholds), whereas Z-scores below “0” indicate hypofunction and therefore a loss of or lower sensitivity of the patient compared with controls (higher thresholds). The QST revealed increased sensitivity to pinprick (MPS) and pressure pain (PPT) and dynamic mechanical allodynia on the affected extremity. There was no loss of detection for thermal or mechanical stimuli. CDT, cold detection threshold; CI, confidence interval; CPT, cold pain threshold; DMA, dynamic mechanical allodynia; HPT, heat pain threshold; MDT, mechanical detection threshold; MPS, mechanical pain sensitivity; MPT, mechanical pain threshold; NRS, numerical rating scale; PHS, paradoxical heat sensitivity; PPT, pressure pain threshold; QST, quantitative sensory testing; TSL, thermal sensory limen; VDT, vibration detection threshold; WDT, warm detection threshold; WUR, wind-up ratio.

Quantitative sensory testing (QST) revealed pinprick and pressure pain hyperalgesia and dynamic mechanical allodynia in a stocking-like manner as well as pressure pain hyperalgesia on the wrist and all finger joints. The somatosensory profile on QST according to the German Research Network of Neuropathic Pain^15^ on the dorsum of the hand is shown in Figure 2. Almost no active movements of the wrist or fingers were possible; central motor signs were not observed. The remaining neurological examination was without pathological findings. The Children's Depression Inventory was normal. The Pediatric Pain Disability Index (PPDI) showed a high degree of disability (PPDI score 4).

**Figure 3.**
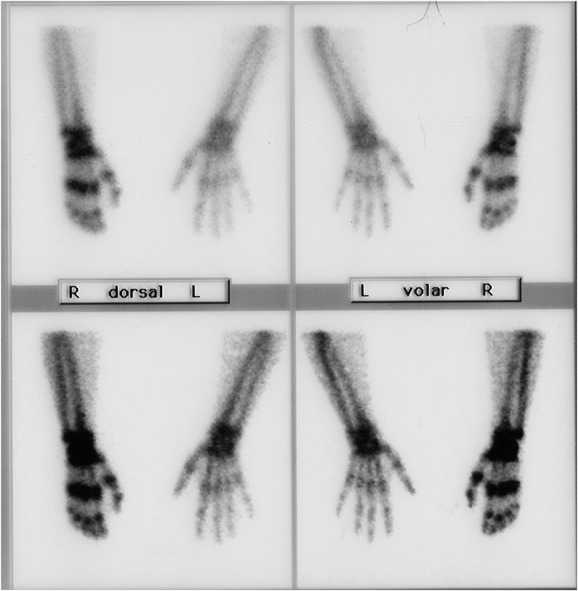
Delayed phase of three-phase bone scintigraphy. The right upper extremity shows a pathological tracer activity in the delayed phase in a strap-like manner on the wrist, the carpometacarpal bones as well as on the proximal and distal small phalangeal joints consistent with the diagnosis of complex regional pain syndrome.

To exclude a psychogenic disuse and support the clinical observation of a reduced joint movement ability, we examined the range of motion during a short narcosis using only propofol without analgesics. This revealed significant side differences of range of motions for the distal joints (wrist: left 70°/0/90°, right 50°/0/60; metatarsophalangeal joints [digiti II, IV]: left 10°/0/95°, right 0/10-15°/40°, proximal interphalangeal joints [digiti II, IV]: left 5°/0/100° right 0/30°/80°, thumb, elbow, and shoulder motion showed no side differences). A TPBS yielded an increase of technetium uptake of the wrist and all small finger joints in the delayed phase in a strap-like manner (Fig. 3). According to this and the other clinical findings, a diagnosis of CRPS I was made.^7^

Multimodal inpatient pain treatment including oral medication (diclofenac, gabapentin), daily physiotherapy, training of pain-related coping strategies such as relaxation techniques, imagination, defocusing, positive reinforcement of pain-free behavior, and operant techniques to reduce anxiety^3,8,9^ for 6 weeks resolved her symptoms substantially. On discharge, she was able to use her hand sufficiently without analgesic treatment. Pain duration improved from constant pain to 30 minutes per day, and mean pain intensity reduced from 8 to 1.5 on the numerical rating scale. The PPDI score reduced to 1 after treatment. The therapeutic effect lasted during the follow-up period over 9 months.

### 1.2. Comment

Although renewed episodes of CRPS in children have been described,^13,16–19^ a recurrent course of disease with only short episodes, without any residues in pain-free episodes and without any new obvious injury in CRPS is rather unusual.

The TPBS showed enhanced periarticular tracer uptake in the small finger joints of the delayed phase which is typical for adult CRPS. This is in contrast to former reports who primarily described a decreased radioisotope uptake in the affected area in pediatric CRPS.^6^ However, clinical signs upon QST in our case resembled adult CRPS^5^ suggesting comparable underlying mechanisms including increased bone metabolism as shown by TPBS.^12^ Accordingly, it has been suggested that CRPS might be overdiagnosed in children, and decreased activity in the delayed phase of TPBS might be due to disuse rather than to CRPS.^10^

However, the short recurrent episodes and the rapid recovering to inpatient treatment strongly contrast the often interminable course of the disease in adults. Within the previous pain episodes diagnostic criteria for CRPS^7^ might have been fulfilled, although signs were overlooked or suspected to be of rheumatic origin. The course of disease suggests that the trauma years ago might have been the initial event of the current CRPS. Small and unrecognized traumas such as riding or playing volleyball might have reactivated the disease. Accordingly, the patient reported that she sometimes had observed a slight pain and swelling after strong exposure. Alternatively, previous pain episodes could be due to an overuse of the extremity and not be interpreted as CRPS. However, the existence of evoked pain accompanied by skin temperature abnormalities and a decreased range of motion is rather unusual for an overuse reaction and strongly supports that the CRPS has been present since the initial event.

Similar to this case, it has been described that a recurrent and migratory CRPS resolved without residues after treatment with physiotherapy and diclofenac in the early phase in a 63-year-old woman^1^ However, the characteristic somatosensory, motor, and TPBS findings suggest that the disease was already present to its full extent.

This case shows that CRPS in children may imitate rheumatologic diseases. Complex regional pain syndrome should be considered if typical signs are present. The diagnosis can be supported by TPBS.

## Conflict of interest statement

R.B. reports current financial interest of affiliation with the following organisations that could be perceived as a real or apparent conflict of interest: Grant/Research Support: Pfizer, Genzyme, Grünenthal; Member of the IMI “Europain” collaboration and industry members of this are: Astra Zeneca, Pfizer, Esteve, UCB-Pharma, Sanofi Aventis, Grünenthal, Eli Lilly, and Boehringer Ingelheim; German Federal Ministry of Education and Research (BMBF): German Research Network on Neuropathic Pain, NoPain system biology. German Research Foundation (DFG). Speaker: Pfizer, Genzyme, Grünenthal, Mundipharma, Sanofi Pasteur, Medtronic, Eisai, Lilly, Boehringer Ingelheim, Astellas, Desitin, Teva Pharma, Bayer-Schering, and MSD. Consultant: Pfizer, Genzyme, Grünenthal, Mundipharma, Allergan, Sanofi Pasteur, Medtronic, Eisai, Lilly, Boehringer Ingelheim, Astellas, Novartis, Bristol-Myers Squibb, Biogenidec, AstraZeneca, Merck, AbbVie, and Daiichi. C.M. reports grants from IMI Europain, grants from German Federal Ministry of Education and Research (BMBF), grants and personal fees from Mundipharma, personal fees from Grünenthal, grants and personal fees from Astellas, personal fees from Epionics, personal fees from AstraZeneca, outside the submitted work. J.G. reports personal fees from Pfizer, TAD Pharma, Glenmark Pharmaceuticals, outside the submitted work. The remaining authors have no conflicts of interests to declare.
